# Effects of Internal Border Control on Spread of Pandemic Influenza

**DOI:** 10.3201/eid1307.060740

**Published:** 2007-07

**Authors:** James G. Wood, Nasim Zamani, C. Raina MacIntyre, Niels G. Becker

**Affiliations:** * National Centre for Immunisation Research and Surveillance of Vaccine Preventable Diseases, Sydney, New South Wales, Australia; † The University of Sydney, Sydney, New South Wales, Australia; ‡ Australian National University, Canberra, Australian Capital Territory, Australia

**Keywords:** Influenza, modeling, travel, pandemic, epidemiology, research

## Abstract

We investigated the capacity of internal border control to limit influenza spread in an emergent pandemic in the context of Australia, a country with a low-population density and geopolitical boundaries that may facilitate restrictions. Mathematical models were used to study the time delay between epidemics in 2 population centers when travel restrictions were imposed. The models demonstrated that population size, travel rates, and places where travelers reside can strongly influence delay. The model simulations suggested that moderate delays in geographic spread may be possible with stringent restrictions and a low reproduction number, but results will be sensitive to the reproduction number and timing of restrictions. Model limitations include the absence of further importations and additional control measures. Internal border control may have a role in protecting domestic centers early in a pandemic, when importations are sparse. Our results may be useful for policymakers.

## Abstract 2

Population size, travel rates, and residence of travelers can aid in determining travel restrictions as a control policy.

Commercial air travel has increased dramatically since the last pandemic of influenza (*1*). The number of international tourist arrivals recorded worldwide in 2004 was 763.2 million; 43% of these arrivals were by air (*2*). This increase in international travel has heightened the risk for the global spread of infectious diseases (*1*).

Long-distance domestic routes also carry high volumes of travelers: an estimated 40.4 million passengers traveled on Australian domestic airlines in the year ending June 30, 2005 (*3*), and 660 million traveled on US domestic airlines during 2005 (*4*). Rapid and accessible long-distance transportation facilitates the geographic spread of diseases, even those, such as influenza, that have a short incubation period (*5*).

If an influenza pandemic emerges, the first attempts to control its spread are likely to be made at its source, as suggested in recent modeling papers (*6*,*7*). However, if these strategies fail, individual governments will need to implement strategies to manage the pandemic when it arrives on their borders. In addition to well-publicized options for control, including antiviral prophylaxis and quarantine (*6*,*7*), travel restrictions, both external and internal, may play a role in reducing the geographic spread of the virus (*8*–*11*)

Restrictions on travel can have a sizeable economic and social impact, as seen in affected nations during the crisis with severe acute respiratory syndrome (SARS). In many countries, stringent travel restrictions will not be feasible because of high population densities and highly connected networks of transportation, infrastructure, and trade. These caveats do not apply to Australia, an island comparable in size to the United States but with a population of only 20 million. This population is concentrated in 5 large cities, along with smaller centers, primarily along its eastern and southern coastlines. These centers are widely separated; travel between them is primarily by air. During the 1918 pandemic, Australia delayed the onset of the pandemic by 1 year by imposing external border control (*12*).

We used mathematical models to make predictions about the effectiveness of travel restrictions and to explore the sensitivity of these predictions to disease and demographic factors. Typically, modeling studies of influenza spread are focused on predicting international or national spread between major hubs on the global air-transportation network (*8*,*9*,*13*), which is certainly important. In contrast, we examine the effects of travel restrictions on 2-city routes with differing characteristics. This simpler setting allows a more detailed exploration of how the delay between epidemics in 2 connected locations depends on travel restrictions, population sizes, travel rates, residence of travelers, and the transmissibility of the influenza virus, with relevance to large and small centers. The analysis assumes case-patients arriving from overseas have a negligible effect, so the results apply primarily during the early stage of a pandemic. Simulating the effect of internal travel restrictions in Australia is relevant to countries with similar demographic characteristics, such as Russia, Canada, and New Zealand. The aims of our analysis were to explore the role of travel restrictions in slowing the geographic spread of an influenza pandemic and to simulate the effects of such restrictions in the context of Australia.

## Methods

**Figure 1:**
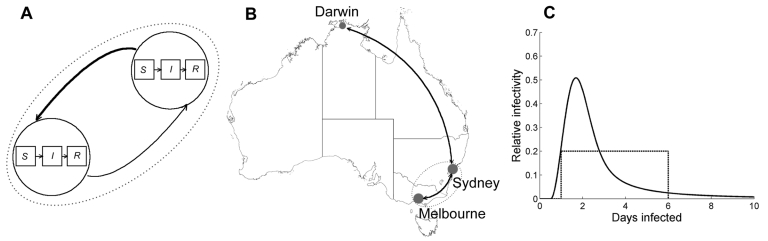
Schematic of travel locations and model. A) Model schematic showing the SIR (susceptible, infectious, and recovered) classes and travel connecting the cities; B) locations of the cities and routes used in the model; C) the form of the 2 infectivity functions used to simulate the infectivity of persons over the course of their infection.

Two simple scenarios (Figure 1A) were used to assess the likely impact of travel restrictions on the spread of a pandemic in Australia. In the first, it was assumed that the initial cases occurred in Sydney. The growth of this epidemic and its resultant spread to Melbourne in the presence of travel restrictions were simulated. This scenario is indicative of the spread to other large centers with similar travel volumes. In the second scenario, the initial case was assumed to occur in Darwin, a smaller Australian city in close proximity to Southeast Asia, and the growth of this epidemic and spread to Sydney were simulated. The Darwin-to-Sydney scenario, with a comparatively low travel volume, represents the situation of containing the epidemic within a smaller town through the use of travel restrictions. Key parameters and assumptions are summarized in Table 1.

**Table 1:** Summary of parameter values, assumptions, and sources used in models of the effect of travel restrictions on pandemic influenza in Australia*

Variable/concept	Value (range)/assumption	Source/interpretation
Reproduction no. (*R*_0_)	1.5–3.5	Mills (*14*)
Infectivity function (ρ)	Flat or peaked†	Longini, Ferguson (*7*,*8*)
Latent period	1 (1–2 in sensitivity analysis) d(s)	Ferguson (*6*)
Infectious period	5 d	Literature suggests 4–7 d in adults (*6*,*7*)
Mixing	Homogenous (within city)	Modeling literature (*15*)
Propensity to travel	Everyone equal	Assumption
Populations	Sydney (4.2 million), Melbourne (3.6 million), Darwin (110,000)	ABS figures (*16*)
Travel rate‡ Sydney ↔ Melbourne (weighted by stay length)	(4.7 × 10^3^, 8.9 × 10^3^)	BTRE figures (*17**,**18*), NSW, and Victoria Tourism reports (*19**,**20*)
Travel rate‡ Sydney ↔ Darwin (weighted by stay length)	(9.2 × 10^4^, 4.4 × 10^3^)	BTRE figures (*17*,*18*), NSW, and NT Tourism reports (*19*,*21*)
Travel restrictions	20%,10%, or 1% of current levels	Assumption
Time between 20 current cases in city 1 and city 2 (*T*_20_)	Random variable (*T*_20_), different for each simulation. Median value over all simulations is given by *m*_20_.	Output variables used to measure effect of travel restrictions

*ABS, Australian Bureau of Statistics; BTRE, Bureau of Transport and Regional Economics; NSW, New South Wales; NT, Northern Territory. †See Figure 1, panel C, for shapes used. ‡This assumes a constant travel rate over the year with no seasonal variation in travel volumes.

### Data

Average daily volumes of domestic air travel between Sydney, Melbourne, and Darwin were obtained from the Australian Domestic Airline Activity report (*17*). Only direct flights were considered. Seasonal variations in the volume of air traffic were not taken into account. Approximately 78% of the traffic from Sydney to Melbourne and 70% of the traffic from the Northern Territory to the eastern Australian states is by air (*18*).

As a separate indicator of travel volumes that incorporates the average length of stay and information on the origin of travelers, we used survey estimates of nights stayed by domestic visitors to the 3 study destinations (Melbourne, Sydney, and Darwin). The data were obtained from the state government tourism websites for Victoria (*20*), New South Wales (NSW) (*19*), and the Northern Territory (*21**)*. Because details on visitor origin were only obtained at the state level, we assumed that each person in that state would make an equal contribution to visitor nights in the destination city. These values were then used to estimate the proportions of the travel volume due to each of the 2 cities on a route and to modify force of infection calculations by incorporating the average length of stay. The travel rates (weighted by length of stay) used in the simulations are provided in Table 1. Demographic data on cities and states were acquired from the Australian Bureau of Statistics population estimates for 2004 (*16*).

### Model Structure

Simulations of influenza epidemics were computed by using a stochastic SIR model, in which the population is separated into 3 mutually exclusive classes: susceptible (*S*), infectious (*I*), and recovered (*R*). A stochastic model can capture random variation near the beginning of an epidemic, when the number of infectious persons is small. Homogeneous mixing is assumed, i.e., all susceptible members of the population in a city are equally likely to be infected by a given infectious person.

A schematic of the model is given in Figure 1B, and the defining equations are presented in the Technical Appendix, part A. The model evolves in discrete time, with the step length equal to 1 day. This time frame accords with real-life epidemics, for which incidence and other epidemiologic data are usually recorded daily. The discrete time structure simplifies the introduction of a variable infectivity profile, incorporating a latent, noninfectious period and a changing degree of infectivity for each person during the course of his or her illness. This feature of the model is supported by virus-shedding studies (*22*) and enables us to contrast the effect of a highly peaked infectivity profile, similar to that used by Ferguson et al. (*6*), with the effect of a constant infectivity profile (*7*), as depicted in Figure 1C.

A key factor governing the effectiveness of our travel restrictions is the average doubling time of the attack rate during the early stages of the epidemic, when growth is exponential. The doubling time is determined by the basic reproduction number (*R*_0_), defined as the average number of secondary infections due to a single primary infected person in a completely susceptible population, and the form of the infectivity profile. The infectivity profile primarily influences the growth rate through the mean time (or serial interval) between cases: ≈2.8 days for the peaked infectivity function and 4 days for the flat infectivity function used here. The doubling time depends linearly on the serial interval so that epidemics that use the peaked infectivity profile double in size almost 1.5× as quickly as epidemics that use the flat infectivity profile, for the same value of *R*_0_. The infectivity profile and *R*_0_ depend on properties of the pathogen and on social, environmental, and genetic factors.

Although influenza appears to be a highly infectious disease, with regular winter epidemics, this is largely due to its short incubation period and genetic drift, which nullifies preexisting immunity. Thus, literature estimates of the effective reproduction number for influenza are typically <4 (*14*) (whereas for measles *R*_0_ is 20 [*15*]), although in localized outbreaks it can be considerably higher (*23*). We take *R*_0_ to be in the range 1.5–3.5, which corresponds to attack rates of 58%–97% (including subclinical infections) in a population without prior immunity or behavioral changes in response to the pandemic.

The total period of infection, including latent period, was assumed to be 6 days (*7*). For each infectivity profile, the latent period was ≈1 day, which is at the low end of literature estimates (other researchers have used values of 1–4 days [*8*,*9*,*24*]). Spread from city to city is incorporated by assuming that each person is equally likely to travel; the daily travel rates were estimated from the data sources described above. This assumption was pessimistic, since symptomatic infected persons may not travel, but it did not greatly influence the results (Technical Appendix, part A).

Travel restrictions were implemented as a reduction of the rate of all forms of travel. For this analysis, reductions of 80%, 90%, and 99% were compared with the base case of unrestricted travel. The values of 80% and 90% might be realistic reduction targets, whereas the value of 99% indicates what near-perfect compliance might achieve. Travel restrictions were switched on in the model at some time (measured in weeks) after the initial case occurred and remained on for the rest of the simulations.

The principal measure used in this analysis for gauging the effect of travel restrictions is *T*_20_, the delay between the epidemic’s becoming established in city 1 and taking off in city 2. We considered the outbreak to have taken off in a city once there were 20 current infectious cases—hence, the notation *T*_20_ for the delay between the epidemics. This choice conveniently limited comparisons to simulated epidemics that do take off. Since the model is stochastic, *T*_20_ is random, and the results shown in the graphs are for *m*_20_, the median value for outbreaks that take off. Ranges, when given, cover 90% of outbreak simulations.

The simulations were run with MATLAB version 7.04 (The MathWorks, Natick, MA, USA) with Poisson random variables simulated by the *poissrnd* function in version 5.02 of the Statistics Toolbox (MathWorks). Our results are based on 10,000 runs of the model.

Motivated by the results of the simulation study, we then analyzed the effects of city size and travel rates by using a deterministic approximation of the above model (details given in the Technical Appendix, part B). This approximation has the advantage of being much simpler to use in analyzing sensitivity to these factors, while reproducing the average behavior of the stochastic model.

## Results

### Scenario 1 (Sydney to Melbourne)

**Figure 2:**
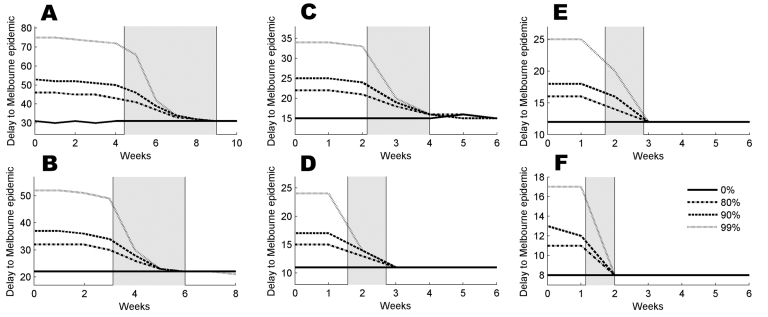
For an epidemic beginning in Sydney, the value of the median time delay, *m*_20_, in the presence of travel restrictions applied at a delay of 0–6 weeks (10 and 8 weeks in [A] and [B], respectively). Assumptions are A) reproduction number (*R*_0_) = 1.5, constant infectivity profile; B) *R*_0_ = 1.5, peaked infectivity profile; C) *R*_0_ = 2.5, constant infectivity profile; D) *R*_0_ = 2.5, peaked infectivity profile; E) *R*_0_ = 3.5, constant infectivity profile; F) *R*_0_ = 3.5, peaked infectivity profile. The gray panes cover the periods when the epidemic grows from 20 to 1,000 infected people in Sydney; dotted, dashed, dash-dotted, and solid lines correspond to 99%, 90%, 80% and no travel restrictions, respectively**.**

The median and mean numbers of days until there are 20 infectious persons in Sydney for an epidemic that began with 1 infectious person in Sydney on day 0 are presented in Table 2. Figure 2 illustrates how *m*_20_, the median time between the day when the number of infected persons first reached 20 in Sydney and the day when the number of infected persons first reached 20 in Melbourne, depends on *R*_0_, the form of the infectivity profile, and the timing and severity of travel restrictions.

**Table 2:** No. days for an influenza epidemic beginning in Sydney to total 20 currently infectious cases*

*R*_0_	Constant infectivity profile†			Peaked infectivity profile†		
	Median, d	90% range, d	Mean, d	Median, d	90% range, d	Mean, d
1.5	24	13–46	25.9	15	8–31	16.4
2.5	12	8–21	13.0	8	5–14	8.4
3.5	9	6–14	9.5	6	4–10	6.2

*R_0_, reproduction number. †The constant infectivity profile assumes that a person is equally infectious throughout their infectious period; the peaked infectivity profile assumes that they are most infectious early in the infectious period (see Figure 1, panel C, for the profiles used).

Each of the graphs covers 1 of the 6 combinations of the 3 values of *R*_0_ and 2 infectivity profiles. The 4 curves shown on each graph describe the median values for each of the 4 levels of travel restrictions (none, 80%, 90%, and 99%), applied at delays from importation of the first case from 0 to 6 weeks (8 weeks for *R*_0_ = 1.5). The gray panes highlight the time during which the epidemic grows from 20 to 1,000 cases in Sydney.

**Figure 3:**
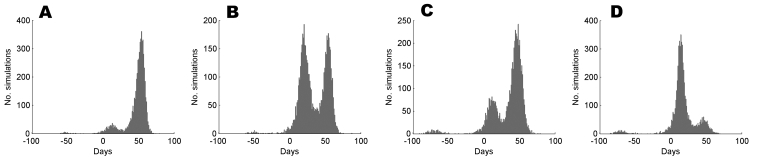
Distributions, based on 10,000 simulations, of the time delay, *T*_20_, given reproduction number (*R*_0_) = 1.5 and the peaked infectivity function, with 99% travel restrictions imposed in scenario 1 (A) and (B) and scenario 2 (C) and (D). Scenario 1 simulates an epidemic beginning in Sydney and spreading to Melbourne. In scenario 2, the epidemic begins in Darwin and spreads to Sydney. In (A) and (C), the restrictions are imposed after 2 weeks; in (B) and (D), they are imposed after 4 weeks**.**

The travel restrictions are most effective for the optimistic assumption *R*_0_ = 1.5 and constant infectivity (Figure 2A). Figure 2B and C more closely resemble the epidemic growth rates used in recent modeling papers (*6*,*7*). In Figure 2B (*R*_0_ = 1.5, peaked infectivity), an increase in *m*_20_ from 22 to 32 days is seen for 80% restrictions, with a further increase to 52 days for 99% restrictions, if applied immediately. These improvements appear robust for delays of up to 4 weeks, but in fact a sizeable proportion of the simulations have spread to Melbourne by this point. This effect is illustrated in Figure 3A and B, in which we compare the full distribution of *T*_20_ in the presence of 99% travel restrictions applied at the 2- and 4-week marks, respectively. Both distributions are bimodal, but in Figure 3B, the first mode is substantial. This difference arises because a large proportion of simulated outbreaks spread to Melbourne between the 2- and 4-week marks for this combination of disease parameters, a finding that emphasizes that timing can be critical for the success of travel restrictions. Under the pessimistic assumption of *R*_0_ = 3.5 and peaked infectivity, the impact of travel restrictions is muted, and a delay of just 2 weeks renders the restrictions ineffective.

### Scenario 2 (Darwin to Sydney)

**Figure 4:**
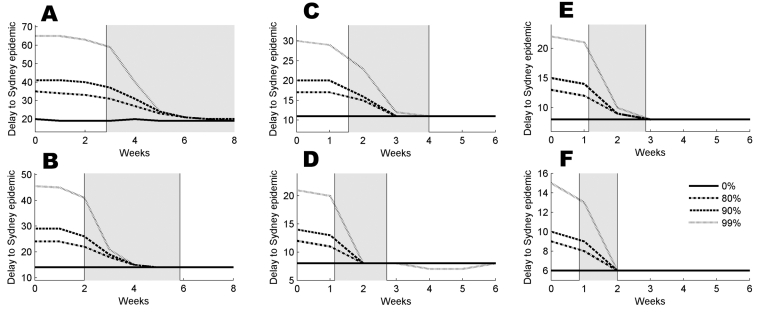
For an epidemic beginning in Darwin, the value of the median time delay, *m*_20_, in the presence of travel restrictions applied at a delay of 0–6 weeks (8 weeks in [A] and [B], respectively). Assumptions are (A) reproduction number (*R*_0_) = 1.5, constant infectivity profile; (B) *R*_0_ = 1.5, peaked infectivity profile; (C) *R*_0_ = 2.5, constant infectivity profile; (D) *R*_0_ = 2.5, peaked infectivity profile; (E) *R*_0_ = 3.5, constant infectivity profile; (F) *R*_0_ = 3.5, peaked infectivity profile. The gray panes cover the periods when the epidemic grows from 20 to 1,000 infected people in Darwin. Dotted, dashed, dash-dotted, and solid lines correspond to 99%, 90%, 80%, and no travel restrictions, respectively.

For an epidemic originating in Darwin, the median times until there are 20 infectious persons in Darwin are almost identical to those for scenario 1 (Table 2), although the 90% ranges are a little wider. In this scenario, *m*_20_ is the median time between the first day on which there are 20 infected persons in Darwin and the first day on which there are 20 currently infected persons in Sydney. The effects of *R*_0_, the infectivity function, and the delay in and severity of travel restrictions are captured in Figure 4.

These results, presented in the same format as Figure 2, show 2 key differences from those in scenario 1. The median delay, *m*_20_, is shorter in scenario 2, given the same combination of disease parameters, as is the time interval over which restrictions can be applied effectively. This finding appears counterintuitive because the volume of travelers on the Darwin-to-Sydney route is much smaller than that on the Sydney-to-Melbourne route.

By using the simpler model described in the Technical Appendix part B, we performed a sensitivity analysis (Appendix Figure) on the effect of city size and travel rates on epidemic spread. This analysis implies that in scenario 2, in which there is a large difference in population size (Darwin:Sydney ≈1:40), infection of susceptible travelers from Sydney is the primary reason for the rapid intercity spread, despite the lower rate of travel for Sydney residents on this route. The Appendix Figure, panel B, shows that this effect would be reduced if the rate at which Sydney residents travel to Darwin were much lower than that for Darwin residents traveling to Sydney. Such a reduction could be achieved by applying tighter restrictions on Sydney-based travelers.

The ratio of city populations also influences the time interval when restrictions can be applied effectively. Travel restrictions were less effective if applied after the time at which there were 20 current cases in Darwin (Figure 4). This feature was illustrated by Figure 3C and D which show the full distributions of *T*_20_ for 2- and 4-week delays in restrictions, respectively. For a 2-week delay (Figure 4C), most outbreaks were delayed but a sizeable minority were not. A 4-week delay (Figure 4D) nullified any impact of the restrictions for this scenario. If, however, travel restrictions were applied immediately after the first case was detected, the increase in *T*_20_ due to restrictions was almost identical to the increases described in scenario 1.

Now consider a situation in which a small isolated center (town A, population 1,000) attempts to remain pandemic free. Let us assume that on any given day, *N* visitors stay in the town, and *N* town members visit pandemic-affected regions. A simple stochastic model of disease spread (Technical Appendix, part D) can predict the probability that the outbreak can be kept out of town A in terms of *N* and *R*_0_ (Technical Appendix part D). Predictions from this model agree well with simulations, as shown in Appendix Figure, panel C. These results indicate that travel restrictions are likely to prevent an outbreak if *N* is reduced to ≈1/10 per day.

### Sensitivity to Other Factors

The sensitivity of the results to the duration of infection and form of the infectivity function were entirely a result of the change in the epidemic growth rate. If, for example, an additional day of latent infection were added, then the delays in spread, when the flat and peaked infectivity functions were used, were ≈25% and ≈37% longer, respectively, which is a considerable effect. However, epidemic growth rates in past pandemics are typically not consistent with longer latent periods and low values of *R*_0_, so these additional delays should be viewed with caution.

The sensitivity to the estimated travel volumes was relatively weak: increasing or reducing travel by a factor of 2 in each direction increases or reduces the delay by 1.5–7 days, and 4.5 or 2.5 days as compared to data in Figures 2 and 4, respectively. These results are consistent for both scenarios.

## Discussion

The simulations we describe showed that although travel restrictions might delay the spread of an influenza epidemic between 2 cities by several weeks, this delay is highly sensitive to assumptions about the transmissibility of the influenza virus. A more surprising result is that the delay is also sensitive to the ratio of city sizes, differences in travel rates, and the originating city. In particular, the modeling suggests that if the epidemic begins in a smaller town, restricting visitors from entering or leaving that town is important.

Moderate delays in the pandemic could be achievable when the epidemic growth rate is low. The growth rate can be estimated from case counts during an epidemic and used in a simple formula to predict the delay due to travel restrictions (Technical Appendix, part D). These predictions could provide practical estimates of the benefits of longer term travel restrictions based on the first clusters of cases during an outbreak. For smaller communities with low travel rates, the probability of preventing an outbreak can also be estimated ( Technical Appendix, part D), with good agreement with the results of our simulations (Appendix Figure, panel C). If the estimated growth rate is high (e.g., assumptions used in Figures 2 and 4 with *R*_0_ = 3.5, peaked infectivity), the additional median delay between 20 cases occurring in city 1 and 20 cases occurring in city 2 might be just 3 days, providing little benefit from longer term implementation of travel restrictions

Our results do not account for additional importations. Thus, they are most applicable to the arrival of a pandemic in Australia, while the pandemic outside Australia remains contained or border control is effective. Our simulated delays will be overestimates if additional importations are substantial. Another concern is that stringent travel restrictions may be required for several weeks to maximize delays in spread. Inevitably, such restrictions would cause economic and social disruption, which must be balanced against any benefits from delaying the domestic spread of an epidemic.

If combined with restrictions on overseas travel, restrictions on internal travel may have a role in pandemic control, even for major centers. However, the economic impact of restrictions in major centers could be enormous, with severe consequences for service and travel industries, as seen in the SARS crisis (*25*), and the potential to affect trade and other sections of the economy. Some of the benefits and costs of reduced travel may also accrue without restrictions, with persons avoiding travel because of perceived risks. Our modeling suggests that travel restrictions could have a greater effect in more isolated communities that lack international ports.

The travel restrictions we discussed have been examined in isolation, without consideration of other disease control measures. Other measures could lower the effective value of the reproduction number, or even curtail the epidemic; in these circumstances, reducing all travel by only 80% might be beneficial. Alternatively, if the *R****_0_***, is much higher than used here (*23*), internal travel restrictions would be ineffective. Limitations of our modeling approach are summarized in Table 3.

**Table 3:** Limitations and effects of modeling effects of border control on pandemic influenza, Australia

Limitations	Effects
Reproduction number (*R*_0_) and infectivity function for pandemic influenza are unknown.	Larger *R*_0_ and a shorter average time between infections would reduce effectiveness of restrictions.
Further importations not considered.	Frequent importations would greatly reduce benefits of internal restrictions for cities with international airports or ports.
Other control measures (pharmaceutical and social distancing) are not considered.	Reductions in transmission would increase effectiveness of restrictions.
Heterogeneous mixing and travel patterns are not considered.	Heterogeneity could increase or reduce delays in epidemic spread. For example, high transmission among infrequent travelers (e.g., the elderly, children) would make restrictions more effective.
Travel rates and restrictions are based on air-travel volumes alone.	Restrictions would prevent no more than 80% of travel if non–air travel remains unrestricted, which would considerably reduce effect of restrictions.
Seasonal variation in travel and transmissibility are not considered.	Could lead to less or more effective restrictions if arrival of pandemic is in winter/summer.

The key points in our study are that delays induced by internal border control are strongly influenced by epidemic growth rates and demographic factors such as the relative sizes of cities, travel rates, and the origin of travelers. When used without other control measures, stopping at least 99% of travel would be required to significantly increase time available for vaccine production and distribution. Although any delay in spread might be attractive for logistical purposes, the economic impact of such restrictions may be prohibitive if sustained for more than a few days. In view of these points, the situation in which they might be most applicable for extended use is in the protection of small, relatively isolated centers.

## Supplementary Material

Appendix Figure. A) *T*_20_ (the time between the days on which the number of infected persons first reached 20 in city 1 and first reached 20 in city 2) (now deterministic) for reproduction number *R*_0_ = 1.5 and 6-day duration of infection, when the city size ratio, *η = N*_1_*/N*_2_, is varied. The epidemic is assumed to begin with 1 infectious person in city 1. Travel rates are equal (*A*_1_ *= A*_2_) with *A*_1_ = 1/1,000; solid, dotted, and dashed lines correspond to persons being free to travel, independent of disease state, only susceptible and removed persons traveling, and only infected persons traveling, respectively. B) Persons are free to travel regardless of disease state, with *A*_1_ = 1/1,000, but the reverse travel rate is varied. Solid, dotted, and dashed curves correspond to *η* = 1, 10, and 1/10, respectively. C) Probability of the outbreak spreading to city 2 as travel volume increases, with markers indicating results from simulations and curves from the analytic approximation. D) the analytic formula for effect of travel restrictions is compared with simulations of scenario 1 (an outbreak beginning in Sydney and spreading to Melbourne) by using the flat infectivity function. Horizontal axes are on a log-scale, and the legend in panel C also refers to panel D**.**

Technical Appendix Simulation Model
