# Supplementary figures and images for: Effects of Internal Border Control on Spread of Pandemic Influenza
Source: Emerg Infect Dis. 2007 Jul;13(7):1038–45. doi: 10.3201/eid1307.060740 (PMC2878213; doi:10.3201/eid1307.060740)

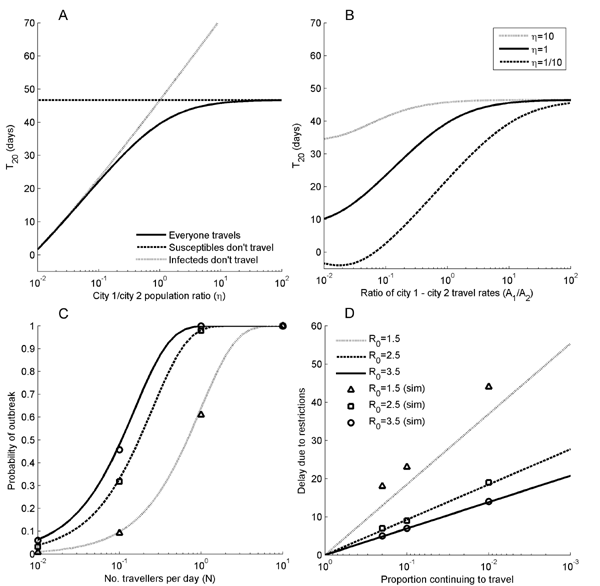

Supplement: Appendix Figure. A) — T20 (the time between the days on which the number of infected persons first reached 20 in city 1 and first reached 20 in city 2) (now deterministic) for reproduction number R0 = 1.5 and 6-day duration of infection, when the city size ratio, η = N1/N2, is varied. The epidemic is assumed to begin with 1 infectious person in city 1. Travel rates are equal (A1 = A2) with A1 = 1/1,000; solid, dotted, and dashed lines correspond to persons being free to travel, independent of disease state, only susceptible and removed persons traveling, and only infected persons traveling, respectively. B) Persons are free to travel regardless of disease state, with A1 = 1/1,000, but the reverse travel rate is varied. Solid, dotted, and dashed curves correspond to η = 1, 10, and 1/10, respectively. C) Probability of the outbreak spreading to city 2 as travel volume increases, with markers indicating results from simulations and curves from the analytic approximation. D) the analytic formula for effect of travel restrictions is compared with simulations of scenario 1 (an outbreak beginning in Sydney and spreading to Melbourne) by using the flat infectivity function. Horizontal axes are on a log-scale, and the legend in panel C also refers to panel D. [file 06-0740_appF-s1.gif]
