# Supplementary material for: Effects of Internal Border Control on Spread of Pandemic Influenza
Source: Emerg Infect Dis. 2007 Jul;13(7):1038–45. doi: 10.3201/eid1307.060740 (PMC2878213; doi:10.3201/eid1307.060740)
Supplement: Technical Appendix — Simulation Model [file 06-0740_Techapp-s2.pdf]

Appendix

(A) Simulation Model

The model equations for a single city are given by

$$\begin{aligned} S(t+1) &= S(t) - X(t), \\ i(t+1) &= X(t), \\ R(t+1) &= i(t-D+1), \\ I(t+1) &= \sum_{d=0}^{D-1} i(t-d+1), \end{aligned} \tag{1}$$

where $i(t)$ denotes the number of new infected persons on day t and is determined by the Poisson random variable $X(t)$ with mean $\mu = S(t)p(t)$. Here,

$$p(t) = 1 - \prod_{d=0}^{D-1} \left(1 - \tfrac{R_0}{N}\right)^{\rho(d)i(t-d)} \tag{2}$$

is the probability on day t of a given susceptible person becoming infected. This equation corresponds to an illness with total infection period of D days and a varying degree of infectivity $\rho(\tau)$ over the course of each infection. Note that $\tau = 0, 1, \ldots, D\text{-}1$, and $\Sigma\rho(\tau) = 1$.

Multiple cities are accommodated by introducing a pair of labels to indicate a person's city of origin and destination as well as a rate of travel A_{kl} from city k to city l per day. The situations considered in this article involve only 2 cities, with just 2 rates of travel, A_{12} and A_{21}. We set $A_{12} \equiv A_1$ and $A_{21} \equiv A_2$, for convenience. The epidemic equations for city 1 become

$$\begin{aligned} S_1(t+1) &= S_1(t) - X_1(t), \\ i_1(t+1) &= X_1(t), \\ R_1(t+1) &= i_1(t-D+1), \\ I_1(t+1) &= \sum_{d=0}^{D-1} i_1(t-d+1). \end{aligned} \tag{3}$$

Here, $X_1(t)$ is a Poisson random variable with mean $\mu_1(t) = S_1(t)[A_1\, p_2(t) + (1\text{-}\, A_1)p_1(t)]$, where $p_1(t)$ is given by

$$p_1(t) = 1 - \prod_{d=0}^{D-1} \left(1 - \tfrac{R_0}{N_1}\right)^{\rho(d)[(1-A_1)i_1(t-d)+A_2 i_2(t-d)]}. \tag{4}$$

Note that the equations for city 2 and $p_2(t)$ can be obtained by replacing the label 1 with 2 and vice versa in the previous 2 equations.

The sensitivity of the results to the assumption that all infected persons travel can be tested by making an optimistic assumption that two thirds of infected persons are symptomatic and do not travel. This leads to an additional median delay of between 2 days (reproduction number $R_0 = 3.5$, peaked infectivity) and 11 days ($R_0 = 1.5$, flat infectivity) if applied to infections acquired while a person is at home or traveling. If, however, one assumes that persons infected while traveling would return home, then the delay is less significant, 1–4 days in scenario 1 and 0–1 days in scenario 2.

(B) Simple Model in Continuous Time

A simpler deterministic model for the infected persons is used to analyze differences in rates of travel on a single route. If we consider only the early stages of the epidemic, well before the peak, then the number of susceptible persons is approximately equal to the total population. The epidemic equations then reduce to the pair of coupled, linear ordinary differential equations that describe the change in the number of infected persons over time:

$$\begin{pmatrix} \dot{I}_1 \\ \dot{I}_2 \end{pmatrix} = \beta \begin{pmatrix} (1-A_1)^2 + \eta\phi_+ A_1^{\ 2} - \gamma/\beta & A_2(1-A_1) + \eta\phi_+ A_1(1-A_2) \\ A_1(1-A_2) + \phi_- A_2(1-A_1)/\eta & (1-A_2)^2 + \phi_- A_2^{\ 2}/\eta - \gamma/\beta \end{pmatrix} \begin{pmatrix} I_1 \\ I_2 \end{pmatrix} \qquad (5)$$

where β is the effective contact rate, 1/γ is the average duration of infection, η is the population of city 1 divided by the population in city 2, A_1 and A_2 are the travel rates from city 1 to city 2 and vice versa, and ϕ_+ and ϕ_- are population modifiers due to travel, with definitions

$$\phi_+ = 1/(1 - A_2 + \eta A_1), \ \phi_- = 1/(1 - A_1 + A_2/\eta). \qquad (6)$$

Symbolically, the solution to these equations can be written as

$$\begin{pmatrix} I_1(t) \\ I_2(t) \end{pmatrix} = \exp(\beta\hat{A}t) \begin{pmatrix} I_1(0) \\ I_2(0) \end{pmatrix},$$

$$\text{where } \hat{A} = \begin{pmatrix} (1-A_1)^2 + \eta\phi_+ A_1^{\ 2} - \gamma/\beta & A_2(1-A_1) + \eta\phi_+ A_1(1-A_2) \\ A_1(1-A_2) + \phi_- A_2(1-A_1)/\eta & (1-A_2)^2 + \phi_- A_2^{\ 2}/\eta - \gamma/\beta \end{pmatrix}. \qquad (7)$$

When susceptible persons are prevented from traveling, this matrix simplifies to the form

$$\hat{A}_S = \begin{pmatrix} 1 - A_1 - \gamma/\beta & A_2 \\ A_1 & 1 - A_2 - \gamma/\beta \end{pmatrix}, \qquad (8)$$

while in the absence of travel by infected persons, it can be expressed as

$$\hat{A}_I = \begin{pmatrix} 1 - A_1 - \gamma/\beta & A_1 \eta \phi_+ \\ A_2 \phi_- / \eta & 1 - A_2 - \gamma/\beta \end{pmatrix}. \qquad (9)$$

Although the equations for the time to spread can be written in analytic form, a numerical solution is required, and hence the MATLAB (The MathWorks, Natick, MA, USA) function *fsolve* is used to compute the solutions graphed in the Appendix Figure panel A.

(C) Dependence on Origin of Travelers and City Size

The dependence on these factors can be studied more generally in terms of differences in population size and travel rates by using the above deterministic version of the stochastic model used in simulations. In this context, T_{20}, the time between the days on which the number of infected persons first reached 20 in city 1 and first reached 20 in city 2, is no longer random. In Figure panel A, the effect on T_{20} of varying the city 1/city 2 population ratio (η) is graphed (assumptions are that the city 1/city2 travel rates are equal, with $R_0 = 1.5$, a 6-day infection period, and the epidemic beginning in city 1). The solid curves show that the delay in epidemic spread increases noticeably as the size of city 1 increases in comparison to city 2. Figure panel B explores the effect of differences in the ratio of city 1/city 2 travel rates for 3 different city population ratios ($\eta = 1$, 1/10, and 10) and shows that this factor can also strongly influence T_{20}.

A better understanding of the effect of city size ratio on T_{20} is obtained by "switching off" the travel of infected persons or, alternatively, susceptible persons in the model. Switching off travel by susceptible persons implies that disease can only spread through the movement of infected persons from city 1 to city 2. Since the ratio, η, does not alter the average number of infections per infectious person (R_0), it should not influence the delay in intercity spread if susceptible persons do not travel. The dashed line in Figure panel A confirms this prediction. If, however, only susceptible persons can initiate journeys, then the disease can only move from city 1 to city 2 through susceptible persons from city 2 acquiring the infection in city 1 before returning to city 2. The ratio of city sizes ought to be important in this instance because the risk of acquiring infection from a single infected person is inversely proportional to the size of the population. This effect is apparent from the dotted line in Figure panel A, with T_{20} increasing linearly with log(η) for all but very small values of the variable.

(D) Analytic Approximations

The effect of travel restrictions can be approximated in terms of the initial growth rate, r, of the epidemic as $T_{p=0.99} = \log(100)/r$, for 99% restrictions and similarly for other levels of travel restriction. The time taken to reach 20 cases can be used to estimate r, and in fact, we can re-express T_p by using this time: $T_{p=0.99} = T_{20}*\log(100)/\log(20)$. In practice, estimates based on T_{20} can be somewhat inaccurate for low values of R_0 (Figure panel D).

The probability of an outbreak spreading from city 1 to city 2, as described by the simulation model above, can be approximated by using extinction probabilities for branching processes (*1*). If, over the course of an outbreak, an attack rate, AR, occurs in the source region, then with N visitors in town A per day and N citizens of town A visiting the source region, the probability of an outbreak occurring is

$$p_A \approx 1 - \exp(-2N(1-q)AR\,R_0), \qquad (10)$$

where q is the extinction probability for a Poisson branching process with mean R_0. As is seen in Figure panel C, where $AR \approx (1-q)$, this approximates the simulation model very well.

Reference

1. Grimmett GR, Stirzaker DR. Probability and random processes, 3rd ed. Oxford (UK): Oxford University Press; 2001.

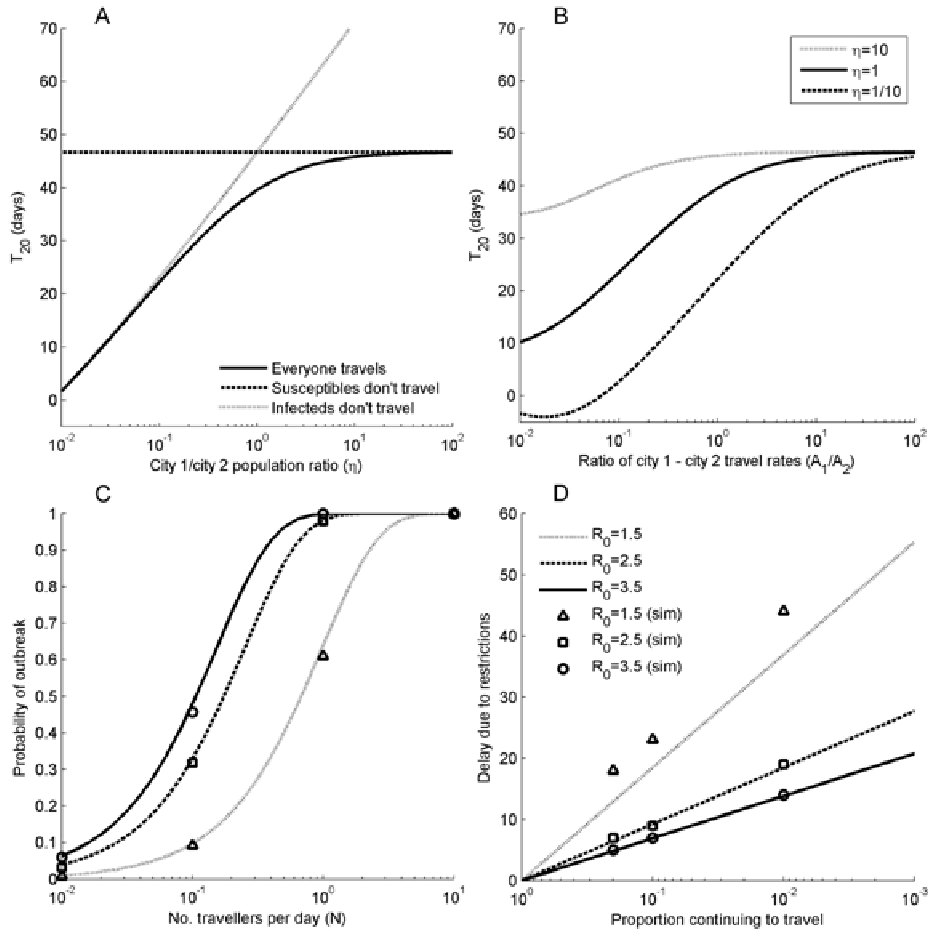

Appendix Figure. A) T_{20} (the time between the days on which the number of infected persons first reached 20 in city 1 and first reached 20 in city 2) (now deterministic) for reproduction number R_0 = 1.5 and 6-day duration of infection, when the city size ratio, $\eta = N_1/N_2$, is varied. The epidemic is assumed to begin with 1 infectious person in city 1. Travel rates are equal ($A_1 = A_2$) with A_1 = 1/1,000; solid, dotted, and dashed lines correspond to persons being free to travel, independent of disease state, only susceptible and removed persons traveling, and only infected persons traveling, respectively. B) Persons are free to travel regardless of disease state, with A_1 = 1/1,000, but the reverse travel rate is varied. Solid, dotted, and dashed curves correspond to η = 1, 10, and 1/10, respectively. C) Probability of the outbreak spreading to city 2 as travel volume increases, with markers indicating results from simulations and curves from the analytic approximation. D) the analytic formula for effect of travel restrictions is compared with simulations of scenario 1 (an outbreak beginning in Sydney and spreading to Melbourne) by using the flat infectivity function. Horizontal axes are on a log-scale, and the legend in panel C also refers to panel D**.**
